# Mixtures of Two Bile Alcohol Sulfates Function as a Proximity Pheromone in Sea Lamprey

**DOI:** 10.1371/journal.pone.0149508

**Published:** 2016-02-17

**Authors:** Cory O. Brant, Mar Huertas, Ke Li, Weiming Li

**Affiliations:** Department of Fisheries and Wildlife, Room 13 Natural Resources Building, 480 Wilson Road, Michigan State University, East Lansing, MI, 48824, United States of America; INRA-UPMC, FRANCE

## Abstract

Unique mixtures of pheromone components are commonly identified in insects, and have been shown to increase attractiveness towards conspecifics when reconstructed at the natural ratio released by the signaler. In previous field studies of pheromones that attract female sea lamprey (*Petromyzon marinus*, L.), putative components of the male-released mating pheromone included the newly described bile alcohol 3,12-diketo-4,6-petromyzonene-24-sulfate (DkPES) and the well characterized 3-keto petromyzonol sulfate (3kPZS). Here, we show chemical evidence that unequivocally confirms the elucidated structure of DkPES, electrophysiological evidence that each component is independently detected by the olfactory epithelium, and behavioral evidence that mature female sea lamprey prefer artificial nests activated with a mixture that reconstructs the male-released component ratio of 30:1 (3kPZS:DkPES, molar:molar). In addition, we characterize search behavior (sinuosity of swim paths) of females approaching ratio treatment sources. These results suggest unique pheromone ratios may underlie reproductive isolating mechanisms in vertebrates, as well as provide utility in pheromone-integrated control of invasive sea lamprey in the Great Lakes.

## Introduction

A common source of conspecific information used in orientation strategies and mate location across the animal kingdom is provided by pheromones, or unique chemical signatures that are released by animals and influence behavior or development of members of the same species [1, 2]. Together, the sending and receiving of pheromones result in movement patterns that reduce the distance between conspecifics across their odor landscape (attraction) and/or maintain individuals in place (arrestant) to gain advantage in mating or feeding [2, 3]. In insects, pheromones that function in sex and aggregation are often comprised of multiple components at specific ratios [4–6]. For pheromones of unique ratios to function as species-specific attractants, a level of discrimination against individual components must occur at the sensory and behavioral level. In fishes, olfactory systems have been shown, via electrophysiological cross-adaptation experiments, to detect and discriminate between compounds with separate receptors [7–9]. Detection and discrimination of individual compounds in phylogenetically similar groups of fishes has been proposed as an adaptation involved in speciation [8]. However, behavioral evidence is rarely presented to evaluate the specificity of pheromone ratios in vertebrates [2].

The olfactory epithelium of the sea lamprey (*Petromyzon marinus*, L.) has been shown to detect and discriminate multiple compounds that are structurally similar [10–12]. Using pheromones for key aspects of their life history [13–16], sea lamprey present a useful vertebrate model for studies regarding pheromone ratio specificity. Sea lamprey begin their single reproductive season by migrating into freshwater tributaries to the Atlantic Ocean (native range), or the Laurentian Great Lakes (invasive range), that are activated with compounds released by stream-residing conspecific larvae [13, 16]. Males often move upstream in greater numbers earlier in the migratory season through April–May, and establish nests in suitable spawning habitat in early June [17, 18]. Upon reaching sexual maturation in streams, males release a pheromone across gill epithelia that contains a main component, 3-keto petromyzonol sulfate (3kPZS).[19, 20]. Synthesized 3kPZS alone draws significant numbers of mature females upstream towards the source [14, 21]. However, 3kPZS as a single component is often less attractive than the whole male odor [14]. Upon analyzing whole male odor (termed spermiated male washings, or SMW, herein), the chemical structure of a new sulfate-conjugated compound, 3,12-diketo-4,6-petromyzonene-24-sulfate (DkPES) was elucidated [22]. Mature females were shown to increase their preference towards mixtures of 3kPZS and DkPES compared to 3kPZS alone. However, many critical issues have not been addressed regarding identity and function of DkPES [22], including chemical synthesis of DkPES and confirmation of the elucidated structure, discrimination of DkPES from 3kPZS by the olfactory epithelia, the orientation mechanisms used by sea lamprey to locate a mixture of these two compounds, and the effective range of ratios between the two pheromone components required for attraction.

This study reports the next step in identifying the function of DkPES as a pheromone component in sea lamprey. Here, we confirm the structure elucidated for DkPES with its synthesized copy, show the olfactory epithelium of sea lamprey discriminates DkPES from 3kPZS, demonstrate that females are attracted to a reconstructed ratio similar to that seen in extracts from mature male sea lamprey [22], and further define the orientation strategies of mature females to the mixture of DkPES and 3kPZS.

## Materials and Methods

### Test Subjects

All surgical and behavioral procedures using sea lamprey presented in this manuscript were approved by the Michigan State University Institutional Animal Care and Use Committee prior to any experimentation (AUF# 05/09-088-00 and 03/11-053-00). Adult sea lamprey used for electro-olfactogram (EOG) recordings were collected in spring 2012 by commercial fishing companies in Lake Huron and transported to United States Geological Survey and Great Lakes Science Center—Hammond Bay Biological Station (HBBS). Sea lamprey were later shipped to Michigan State University, East Lansing, Michigan for EOGs. Migrating adult sea lamprey were captured by the United States Fisheries and Wildlife Service and Department of Fisheries and Oceans Canada from tributaries to Lake Michigan and Lake Huron in May–June 2012, following animal use and care protocols established by those agencies. Live lamprey were transported to HBBS and held in 500–1000 L-capacity flow through tanks until use. Males were kept separate tanks from females and never used for this study. Sea lamprey are not native to the Laurentian Great Lakes and their tributaries. Hence, males and females could not be released together upstream of sea lamprey barriers to reduce the risk of repopulation of the river system.

To produce sexually mature ovulated female (MF) test subjects, lamprey were transferred to acclimation cages constructed of polyurethane mesh and PVC pipe (0.5 m^3^) located in the lower Ocqueoc River, Millersburg, Michigan, to allow natural maturation *in situ*. Sea lamprey were monitored daily for signs of sexual maturation. Briefly, MFs were identified by first checking for secondary sexual characteristics [23], then applying gentle pressure to the abdomen and checking for expression of ovulated oocytes from the cloacal aperture.

### Purity analysis of DkPES

**Fig 1:**
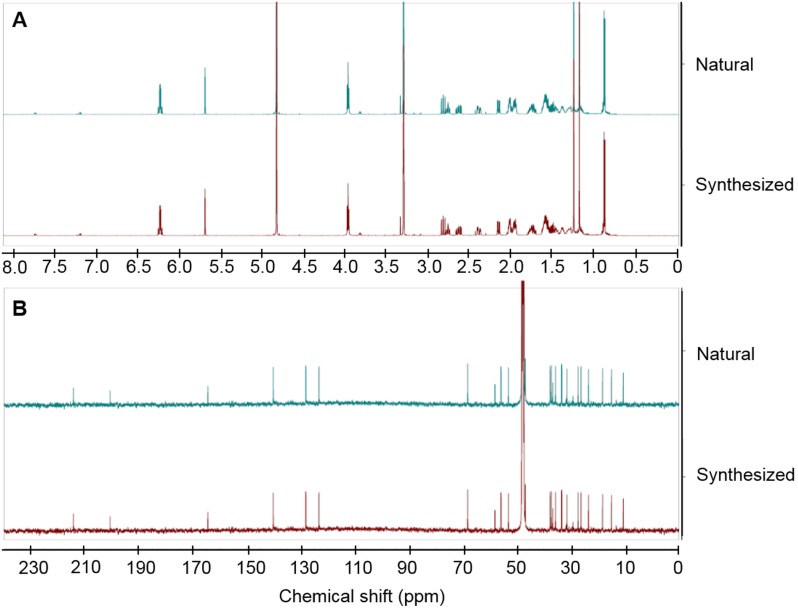
Spectral comparisons of synthesized and natural (purified) DkPES. **(A)** Comparison of ^1^H NMR spectra of natural and synthesized DkPES obtained from 600 MHz NMR spectrometry (Varian Inova) in methanol-*d*_4_. **(B)** Comparison of ^13^C NMR spectra of natural and synthesized DkPES obtained from 600 MHz NMR spectrometry (Varian Inova) in methanol-*d*_4_.

The quality of synthetic DkPES (chemical structure and purity) were examined using a series of chemical analyses by high resolution mass spectrum (HR-ESI-MS). ^1^H, ^13^C NMR spectra, ^1^H-NMR and ^13^C NMR experiments were performed on synthetic DkPES ammonium salt using a Varian Inova 600 MHz NMR spectrometer at the Michigan State University Max T. Rogers NMR facility, East Lansing, MI, USA. Samples (ca. 5.0 mg) were prepared in CD_3_OD and subjected to NMR analysis. The results were compared with original DkPES and displayed in Fig 1.

HR-ESI-MS of synthetic DkPES ammonium salt was performed on a TQ-S TOF LC mass spectrometer (Waters Corporation, Milford, MA, USA). The solution (10 μL) of synthetic DkPES (1.0 μg/ml, MeOH/H_2_O = 1:1, v/v) was injected by auto-sampler. The mobile phase consisted of water (A), and methanol (B). The isocratic gradient (30%A and 70%B) was used as an eluent. The UHPLC effluent was introduced into the mass spectrometer with electrospray ionization in the negative mode. The ESI-MS parameter was set as capillary voltage, 2.60 kV; extractor voltage, 5 V; source temperature, 150°C; desolvation temperature, 500°C; desolvation gas flow, 800 L/h (N_2_, 99.9% purity). Argon (99.9999% purity) was introduced as the collision gas into the collision cell at a flow rate of 0.15 mL/min. Data were collected in centroid mode with a scan range of 50–1000 *m/z*. Data processing was performed on MassLynx 4.1.

DSC were used to evaluate the absolution purity of synthetic DkPES without standard. The DSC experiment was carried out on DSC Q2000 (TA Instrument, New Castel, DE, USA). Sample (ca. 3.2 mg) was set in a Tzero^™^ pan with lid (TA Instrument, New Castel, DE, USA) and stored on an oven by auto-sampler. The temperature ramp was set from 20°C to 250°C, and heating rate was 1.0°C/min. The absolute purity of DkPES was analyzed using TA Instrument’s Universal Analysis 2000 software, provided by the manufacturer.

### Synthesized Pheromone Components

The compound 3kPZS was custom synthesized by Bridge Organics Co. (purity = 97%, Vicksburg, MI) as a white powder salt. DkPES was chemically synthesized by Apeloa Kangyu Pharmaceutical Co. (purity = 97%, Dongyang, Zhejiang, China) as a white powder salt. The synthetic compound exhibited the same spectral characteristics and biological activity as the published natural compound [24]. A 1 mg/mL stock solution of each compound (in 50% methanol:deionized water) was prepared. Stock solutions were stored at -20°C until use.

### Electro-olfactogram (EOG) Recording

Electro-olfactogram recordings were obtained from sea lamprey in spring 2012. Our procedures for EOG are detailed in Li et al. [22]. Briefly, sea lamprey were anesthetized with 3-aminobenzoic acid ethyl ester (100 mg/L; MS222, Sigma-Aldrich Chemical Co.), immobilized with an intra-muscular injection of gallamine triethiodide (3 mg.kg^-1^, in 0.9% saline), and placed in a partially inundated V-shaped Plexiglas cradle. Gills were continuously irrigated with aerated water containing 50 mg/L MS222. The olfactory rosette was surgically exposed and olfactory responses to stimuli were recorded by borosilicate electrodes filled with 0.04% agar in 0.9% saline, connected to solid-state electrodes with Ag/AgCl pellets in 3 M KCl. Electrodes were placed between olfactory lamella (recording electrode) and external skin (reference electrode). Olfactory responses were filtered and amplified by a NeuroLog system model NL102, filtered with a low-pass 60Hz, model NL125 (Digitimer Ltd., Hertfordshire, England), digitized by a Digidata 1550 (Molecular Devices LLC., Sunnyvale, California), and stored on a PC running AxoScope 10.4 software (Molecular Devices LLC).

**Fig 2:**
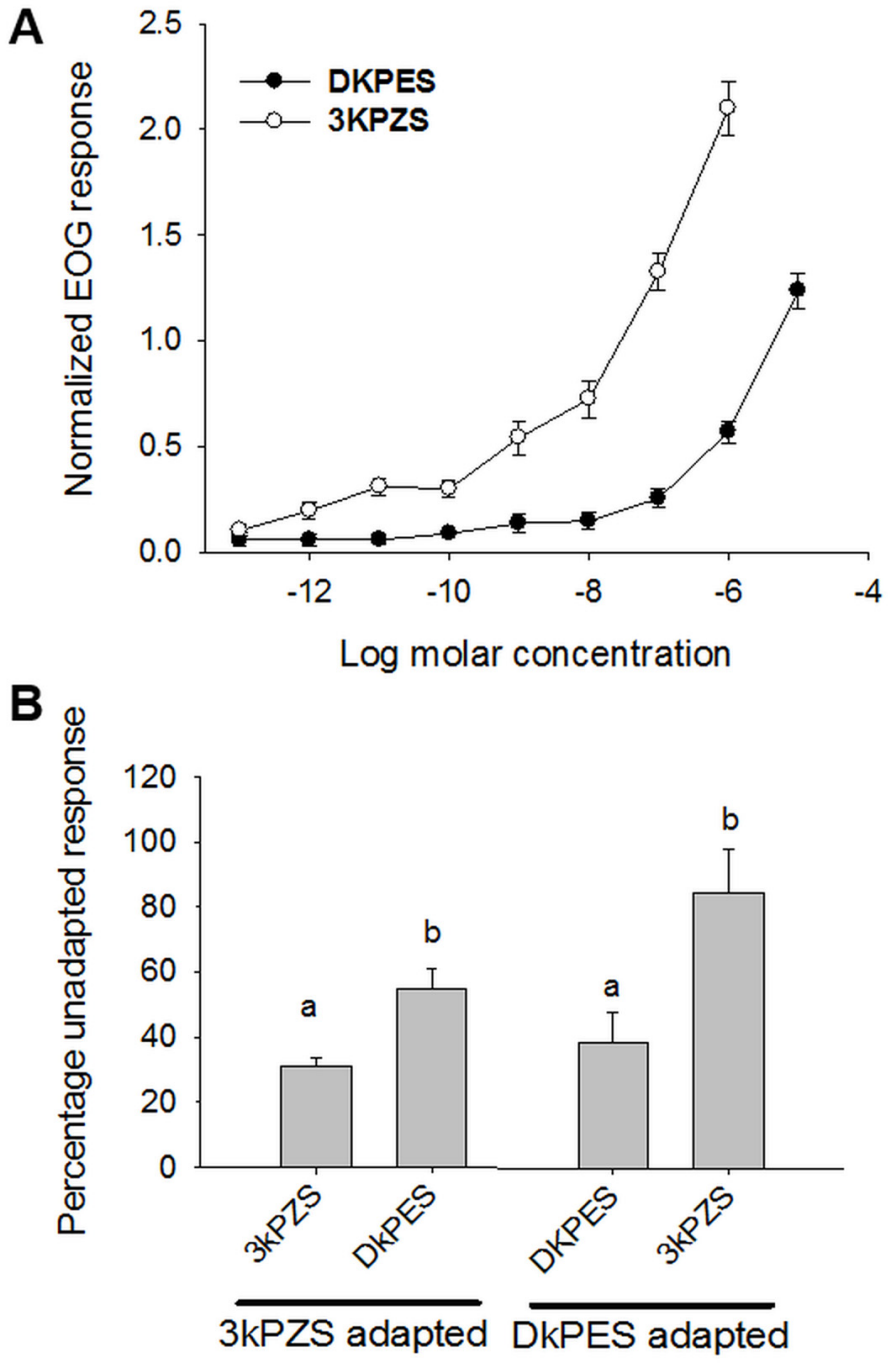
Olfactory detection and discrimination of DkPES and 3kPZS by adult sea lamprey. **(A)** Semi-logarithmic plots of electro-olfactogram (EOG) responses to different concentrations of DkPES and 3kPZS. The response amplitude was corrected for the blank response amplitude and normalized against the response amplitude of a standard odorant, L-arginine at 10^−5^ Molar. **(B)** Differences in DkPES and 3kPZS assessed by cross-adaptation. Percentage unadapted response is the response amplitude to an odorant (treatment) when the olfactory epithelium was pre-adapted to an odorant, expressed as a percentage of the response amplitude to the same testing odorant when the olfactory epithelium was not pre-adapted. 3kPZS adapted and DkPES adapted indicate when the olfactory epithelium was pre-adapted to 3kPZS and DkPES, respectively. Different letters indicate significant differences in responses amplitude with respect to the corresponding adaptation (DkPES: *t* = -3.59, *P* = 0.009 3kPZS: *t* = -4.68, *P* = 0.002. Vertical bars represent one standard error, *n* = 5.

For concentration-response curves, synthesized 3kPZS or DkPES was serially diluted in charcoal filtered water from a 10^−3^ M stock solution, respectively. Responses were measured in mV, blank subtracted and normalized to those of L-arginine at 10^−5^ M (Fig 2A). The detection threshold was defined as the lowest concentration where the EOG response was different from the blank normalized response, and was determined by Student’s *t*-test (*α* = 0.01, with Bonferroni correction). The responses of the lowest five concentrations were used in this analyses, as the responses at higher concentrations showed an apparently different slope on the curve.

Cross-adaptation experiments followed the protocol of Huertas et al. [25]. Dilutions of 3kPZS and DkPES that evoked the same EOG amplitude (10^−9^ M 3kPZS and 10^−7^ M DkPES) were recorded as normal, or the ‘unadapted’ response. The olfactory rosette was continually exposed to unadapted 3kPZS solution for at least 1 min, and the response to a sample of 3kPZS at 2x10^-9^ M was recorded as the ‘self-adapted control’. The responses to a mixture of 3kPZS and DkPES at 10^−9^ M and 10^−7^ M, respectively, was then recorded as the ‘adapted’ response. The amplitudes of self-adapted control and adapted responses were then showed as a percentage of the appropriate unadapted response. The olfactory epithelium was exposed to charcoal filtered water for 10 min, and this process was repeated using DkPES as the adapting solution and 3kPZS and the mixture as stimuli. The sequence of adaptation of 3kPZS and DkPES were randomized. Differences between responses of test odorants recorded before and after the epithelium was adapted to a particular odorant were tested by Student’s *t*-test (*α* = 0.05, Fig 2B).

### Passive Integrated Transponder (PIT) Tagging Procedures

Passive integrated transponder (PIT) tagging procedures for MFs followed Johnson et al. [14]. PIT tags were half-duplex 23 mm glass tags (Texas Instruments, Dallas, TX). Each PIT tag was fitted into a latex sleeve and attached to the mid-dorsal region of each MF using a suture on both sides (Size 3–0, Ethicon Inc., Cornelia, GA). Subjects were also fitted with unique color combinations of ribbon tags (Hallprint, Hindmarsh Valley, South AU) through each dorsal fin to identify individuals for visual tracking during trials. Tagged animals were immediately transferred into aerated holding tanks with a constant flow of Lake Huron water for up to 24 hours, until they were stocked into stream acclimation cages.

### Field Bioassays

**Fig 3:**
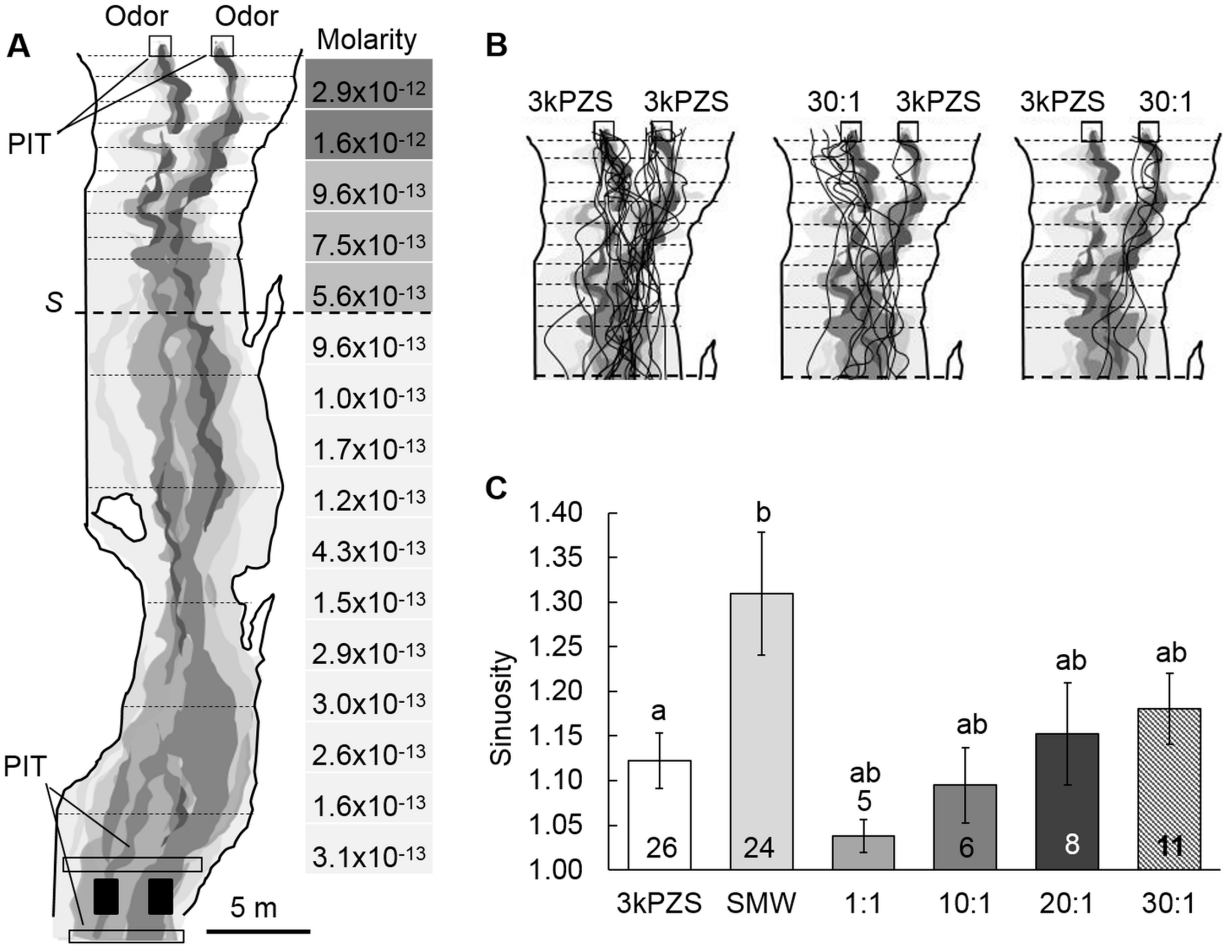
Behavioral responses of mature female sea lamprey to DkPES in a natural spawning stream. **(A)** Schematic of the 45 m-long section of the Upper Ocqueoc River used for field bioassays. Rhodamine dye was administered and sampled at ten points along each transect (transecting dotted lines) to map the odor plume. Average rhodamine dye concentrations every 2 m downstream from the odor sources are shown to scale on the key labeled “Molarity.” The shading of the Molarity key corresponds with the grey shading of the treatment plume, showing “ribbons” of concentrated treatment throughout the center of the stream. Based on rhodamine dye concentrations, it was estimated that treatments became distributed from bank-to-bank at roughly 11 m downstream of the source, indicated at transect *S*. Locations of passive integrated transponder (PIT) antennae are shown. Downstream release cages are indicated with solid black boxes. **(B)** Swim tracks of individual test subjects during 3kPZS (5x10^-13^ M) vs. 3kPZS (5x10^-13^ M) control treatments and 3kPZS (5x10^-13^ M) vs. ratio 30:1 (5x10^-13^ M 3kPZS:1.7x10^-14^ M DkPES) treatments are shown starting at point *S*. **(C)** Mean sinuosity (track length/shortest connecting line) of tracks for each treatment (± 1 SEM) was calculated from point *S* up to adjacent nests (~ 15 m) during treatments: 3kPZS (5x10^-13^ M), spermiated male washings (SMW, applied at 5x10^-13^ M 3kPZS benchmark), ratio 1:1 (5x10^-13^ M 3kPZS:5x10^-13^ M DkPES), ratio 10:1 (5x10^-13^ M 3kPZS:5x10^-14^ M DkPES), ratio 20:1 (5x10^-13^ M 3kPZS: 2.5x10^-14^ M DkPES), and ratio 30:1. Treatments that share a letter are not significantly different (ANOVA and *post-hoc* Tukey’s HSD: *F*_5,74_ = 3.19, *P* = 0.012). The number of responding subjects (*n*) are indicated within each column.

Trials were ran from 12 June– 27 July 2012, a time-frame representative of a typical spawning season for sea lamprey [18, 23], in a 45 m-long stretch of the Upper Ocqueoc River, Millersburg, MI. The field site is consistent with our previous study [22], with slight modification. Two 1 m^2^ nest antennae were placed side-by-side to one another on the upstream end of site, laid flat on the stream bed, 2 m apart from frame to frame. These antennae monitored the proportion of subjects that entered a particular “nest” containing treatments. Downstream 45 m, two aluminum-mesh release cages (0.25 m^3^) equipped with sliding release doors were positioned in the center of the stream channel. A PIT antenna (0.5 m-high x 6 m-long) was positioned 1 m upstream of the release cages and 1 m below release cages (0.5 m-high x 5 m-long) to monitor individuals that exit the cage and move upstream (Fig 3A).

Stream temperatures were recorded at the start of and end of each trial. Treatments were diluted with 20 L of river water in large mixing bins on shore. Bins were kept consistent for each test treatment, and rinsed in the stream several times before each new trial to reduce the potential for contamination during mixing. Each treatment solution was then pumped from bins into the stream at the center of each “nest” antenna at a rate of 167 mL/min (± 3 mL/min) using peristaltic pumps (Cole-Parmer). Trials were a total of two hours long. In the first half-hour of each trial the treatments were administered to the stream while subjects remained in the release cage. At the start of the following 1.5 hours, subjects were released and their movements were monitored with PIT antennae until the trial ended. No animals were recovered from the stream after a trial.

Copper wire was wrapped around each antenna frame twice during the construction of PIT antennae for a more focused read range. Antennae were wired to a multiplexor and data logger in the field for consolidation and storage of data (Oregon RFID, Portland, OR). Antennae were tuned to a detection sensitivity of roughly 0.3 m from the frame edges. Scan frequencies of each antenna were programmed to three scans/sec. Data for each trial were uploaded each day using a hand-held Meazura model MEZ1000 personal digital assistant (Aceeca International Limited, Christchurch, New Zealand).

### Details of Treatments

The desired treatment concentration (molarity) for each trial was estimated based on stream discharge (whole-stream concentration). Stream discharge was estimated every three days, or after every precipitation event, at a fixed location in the stream using a Marsh-McBirney portable flow meter (Flo-Mate 2000, Fredrick, MD) to determine the volume of treatment stock solution to add to mixing bins to maintain consistent concentrations across trials. The volume of stock solution required for each trial was estimated based on the total volume of water that would pass through the system in the allotted trial time (2 hrs), the desired whole-stream concentration (5x10^-13^ M for 3kPZS), and the molecular weight of the compound (490: synthesized 3kPZS). Treatments and desired whole-stream concentrations included: (1) 3kPZS (5x10^-13^ M) vs. 3kPZS (5x10^-13^ M), (2) spermiated male washings (SMW, applied at 5x10^-13^ M 3kPZS benchmark) vs. river water (RW), (3) ratio 1:1 (5x10^-13^ M 3kPZS:5x10^-13^ M DkPES) vs. 3kPZS (5x10^-13^ M), (4) ratio 10:1 (5x10^-13^ M 3kPZS:5x10^-14^ M DkPES) vs. 3kPZS (5x10^-13^ M), (5) ratio 20:1 (5x10^-13^ M 3kPZS: 2.5x10^-14^ M DkPES) vs. 3kPZS (5x10^-13^ M), and (6) ratio 30:1 (5x10^-13^ M 3kPZS:1.7x10^-14^ M DkPES) vs. 3kPZS (5x10^-13^ M). Treatments applied to each “nest” were alternated each trial. Up to two trials were conducted each day depending upon the availability of mature animals. The early trial was conducted from ~0700h–0900h, and a late trial was then run from ~0930h–1130h. Ten PIT-tagged MFs were transferred to respective acclimation/release cages for each trial between 2000–2200h the night prior to experimentation. Subjects were then allowed an acclimation period in the stream for a minimum of 9 hours.

### Swim Track Mapping

Swim tracks were mapped during trials following Johnson et al. [14], with slight modification stated here. The stream section was fixed with transecting strings every 1 meter downstream of “nests” for the first 10 m, and every 5 m down after that until reaching release cages. Each transecting string was divided into equal tenths (of the total stream width). Since each test subject was marked with a unique color combination of ribbon tags, we were able to visually observe and record individuals onto scale maps by hand as they swam upstream. Observers followed each subject until reaching the nests, using transecting strings as reference markers. Only subjects that were observed exiting the release cage were followed. Preference responses for the rest of the subjects that exited unseen by observers were recorded via PIT antennae.

To map the odor plume, rhodamine dye was administered to the stream at our treatment pumping rate (167 mL/min) on July 10^th^, when streamflow was 537 L/sec (which fell into the range of our average stream flow across all trials of 598 ± 76 L/sec). After allowing the dye to be administered for 30 min., rhodamine concentrations were detected and recorded at each sample point (*i*.*e*. every tenth of the stream widths, marked along transecting strings) with a hand-held DataBank datalogger and Cyclops-7 Optical Rhodamine Dye Tracer (Turner Designs, Sunnyvale, CA). All swim tracks were traced onto a digital map using a tablet computer (Lenovo X201 Tablet). Swim tracks and the rhodamine dye plume were both mapped to scale, independently, and tracks were later overlain onto plumes in a double-blind design.

### Statistical Analyses of Behavioral Data

Based on our dye test, it was estimated that the treatment became distributed from bank-to-bank at a point roughly 11 m downstream of the source. This transect was labelled *S* in Fig 3A. Important to note is that *S* did not represent the point at which the treatment was equally mixed in the stream water, as ribbons of concentrated treatment still existed towards the center of the stream (represented by the darker grey areas in Fig 3A). The dye concentration detected at each of ten points along transect *S* averaged 5.6x10^-13^ M, which approached that of our target whole-stream concentration of 5x10^-13^ M. Sinuosity of each swim track was calculated by dividing the track length (starting at transect *S* and ending at the downstream edge of the source antenna) by the length of a straight line connecting the start and end of each track. Transect *S* was chosen for a sinuosity calculation start point because it was the point at which lamprey would begin exposure to a gradual increase in odorant concentration and plume edges that would allow subjects to cast into and out of the plume structure. Since sinuosity values are proportions (*i*.*e*. a value of 1 is a straight line), values were square root-transformed. The Levene’s test for homogeneity of variance was used to examine variance of newly transformed data. Once homogeneity of variance was observed, an ANOVA and *post-hoc* Tukey’s HSD (*α* = 0.05) was conducted for statistical comparisons of sinuosity data across treatments using R-software (R version 2.11.1 Vienna, Austria)

For all PIT telemetry data, movement of subjects during treatment applications were analyzed using a general linear model with a binomial distribution. All subjects were assumed to behave independent from one another as observed from previous field studies with sea lamprey [14, 21, 22]. Binary data evaluated with logistic regression (R version 2.11.1, Vienna, Austria) showed no evidence of overdispersion or nonlinearities in the general linear models with a random effect of trial date and stream temperature [14]. All behavioral statistics reported are two-tailed analyses (*α* = 0.05). Two main binary response variables were examined from PIT data: (1) the distribution of subjects that swam upstream from release cages and did not move back down (*Up*: 1 = hit on upstream release antenna and continued towards nests, 0 = did not hit on upstream release antenna) and (2) of those animals that hit on the upstream antenna, the distribution that entered the “nest” containing the test treatment (*Enter nest*: 1 = hit treatment nest, 0 = hit control nest). Since 3kPZS was administered to both nests during control trials, one nest was randomly assigned for statistical purposes to be the “treatment” nest. The “treatment” nest was randomly chosen to be the right nest, and alternated every trial to follow the same pattern of the other treatments. Each test subject’s unique PIT tag identification number prevented any pseudo-replication from test subjects released during trials.

When a subject entered a nest, observers recorded the amount of time spent inside the 1 m^2^ area (retention, min.) until respective subjects moved on. All retention data was examined for violation of assumptions of normality and homogeneity across variance before further statistical analyses were conducted. Retention data that were not normally distributed or showed heterogeneity across variance were log-transformed. The Levene’s test for homogeneity of variance was used to examine variance of newly transformed data. Once homogeneity of variance was confirmed, an ANOVA and *post-hoc* Tukey’s HSD (*α* = 0.05) was conducted for statistical comparisons of retention across treatments (R version 2.11.1).

## Results

### Spectra of synthesized DkPES match those of purified DkPES

The compound DkPES ammonium salt applied in this study was synthesized by Apeloa Kangyu Pharmaceutical Co. (Dongyang, Zhejiang, China) according to the structure deduced in the preceding study [22]. Differential scanning colorimetry (DSC) analysis [26, 27] of the synthetic product indicated that its purity was 97.0% (S1 Fig). The chemical structure of DkPES was further confirmed by comparison of high resolution mass and NMR spectra of the purified compound [22] and the synthesized copy. The pseudo-molecular formula determined by high resolution mass spectrometry (*m/z* 449.2012 [M—NH_4_]–) of the synthetic product was C_24_H_33_O_5_S, which was in good agreement with the calculated mass (*m/z* 449.1998, ΔmDa 1.4, ppm 3.1, S1 Table). The purified and the synthesized compounds showed overlapped NMR spectra (Fig 1), indicating that they are identical in their chemical structure.

### DkPES is discriminated from 3kPZS in sea lamprey olfactory epithelia

Electro-olfactogram (EOG) recordings showed that both synthesized 3kPZS and DkPES are highly stimulatory for the olfactory epithelia of adult sea lamprey (Fig 2A). At each concentration tested, the amplitude of the olfactory response elicited by 3kPZS was larger than that elicited by DkPES. The threshold of detection for 3kPZS was 10^−13^ M (P<0.001) and for DkPES was 10^−10^ M (P<0.001). The difference in the magnitude and slope in the concentration-response relationships for 3kPZS and DkPES suggested that the olfactory receptor mechanisms for these two compounds may differ [28]. This hypothesis was further supported by a set of cross-adaptation experiments [28], in which preadaptation of the olfactory epithelium to one compound did not suppress the olfactory responses to the other. In particular, when the sensory epithelium was subjected to prolonged perfusion (pre-adaptation) with 3kPZS, the normalized EOG response to DkPES was larger than that to 3kPZS (Fig 2B, Student’s *t*-test, *t* = -3.59, *P* = 0.009). Vice versa, when the epithelium was pre-adapted to DkPES, the EOG response to 3kPZS was larger than the response to DkPES (Fig 2B, *t* = -4.68, *P* = 0.002). These results indicate that the olfactory epithelium distinguished DkPES and 3kPZS by at least two different sets of receptors.

### Females are attracted to the 3kPZS and DkPES mixture at ratios similar to those identified in SMW extracts

A 30:1 ratio of 3kPZS:DkPES was observed in spermiated male wash-water (SMW) extracts by Li et al. [22], and thus the 30:1 ratio was examined along with other ratios during these trials. Swim tracks overlain onto rhodamine plume map suggest that subjects showed a preference for the 30:1 3kPZS:DkPES mixture compared to 3kPZS alone in the adjacent nest (Fig 3B). Sinuosity was highest when subjects were exposed to SMW (data available in S1 Data). Mean sinuosity of swim tracks was lowest during 1:1 mixture treatments, increased up to 30:1 treatments, and peaked during SMW treatments (ANOVA and *post-hoc* Tukey’s HSD: *F*_5, 74_ = 3.19, *P* = 0.012). Specifically, mean sinuosity did not differ between SMW and 30:1 treatments (HSD: *P* = 0.766), yet was lower for 3kPZS treatments compared to SMW treatments (HSD: *P* = 0.023, Fig 3C). Full swim tracks for all treatments can be seen in S2 Fig.

**Fig 4:**
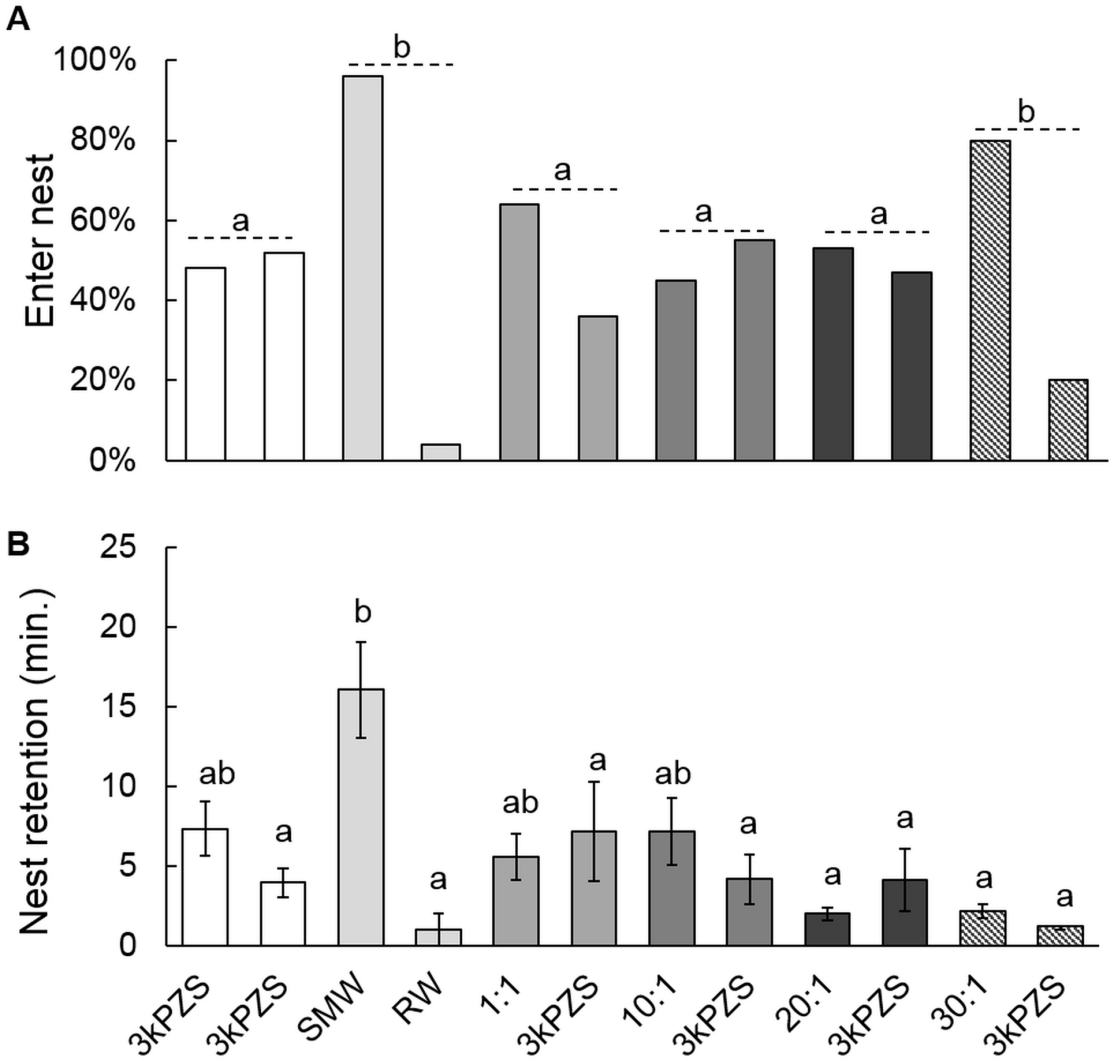
Preference response of mature female sea lamprey to various ratios of pheromone components in side-by-side stream nests. Treatments included: 3kPZS (5x10^-13^ M, *n* = 16) vs. 3kPZS (5x10^-13^ M, *n* = 17), spermiated male washings (SMW, applied at 5x10^-13^ M 3kPZS benchmark, *n* = 26) vs. river water (RW, *n* = 1), ratio 1:1 (5x10^-13^ M 3kPZS: 5x10^-13^ M DkPES, *n* = 16) vs. 3kPZS (5x10^-13^ M, *n* = 9), ratio 10:1 (5x10^-13^ M 3kPZS:5x10^-14^ M DkPES, *n* = 9) vs. 3kPZS (5x10^-13^ M, *n* = 11), ratio 20:1 (5x10^-13^ M 3kPZS: 2.5x10^-14^ M DkPES, *n* = 8) vs. 3kPZS (5x10^-13^ M, *n* = 7), and ratio 30:1 (5x10^-13^ M 3kPZS:1.7x10^-14^ M DkPES, *n* = 12) vs. 3kPZS (5x10^-13^ M, *n* = 3). Dashed vertical grey lines separate pair-wise comparisons. **(A)** Percentage of subjects that entered each treatment nest. Treatments that share a letter are not significantly different (Logistic regression: *X* ^2^_5_ = 25.51, *P* < 0.001). **(B)** Pair-wise comparison of mean (± 1 SEM) retention (min.) of subjects inside respective treatment nests. Treatments that share a letter are not significantly different (ANOVA and *post-hoc* Tukey’s HSD: *F*_11,123_ = 3.55, *P* < 0.001).

From passive integrated transponder (PIT) telemetry data (data available in S1 Data), the proportion of mature female subjects that moved upstream was not different across treatment levels (Logistic regression: *X*^2^_5_ = 5.85, *P* = 0.322, S2 Table). This is likely due to the fact that all treatments contained 3kPZS, a pheromone component known to induce upstream movement of mature female sea lamprey [14]. The proportion of subjects entering the treatment nest (within 0.5 m of the treatment source) during SMW or 30:1 treatments did not differ from on-another (Two-tailed *t*-test: *t* = 1.52, *P* = 0.132), and both showed highest entry rates (*X*^2^_5_ = 25.51, *P* < 0.001, Fig 4A) compared to all other treatments. However, the mean time spent within the nest by mature females differed between SMW and the 30:1 treatments. Specifically, subjects showed higher retention (minutes) inside nests when SMW was administered (*F*_11, 123_ = 3.55, *P* < 0.001) compared to all other treatments. No differences were observed in retention time across all other treatments (see Fig 4B for all statistical comparisons, data available in S1 Data).

## Discussion

This study confirms the structure of DkPES and defines the function of DkPES and 3kPES as a pheromone mixture in sea lamprey. Our previous work suggested that certain ratios of DkPES and 3kPZS may be important for orientation of receiver females to the odorant source, and mature females showed a behavioral preference to the mixture at multiple ratios [22]. In the present study, we confirmed unequivocally the elucidated structure of DkPES through chemical synthesis, showed that DkPES was discriminated from 3kPZS in the olfactory epithelia, and elucidated orientation strategies of receiver females exposed to mixtures of DkPES and 3kPZS in a range of ratios.

From the data on locomotion patterns of mature females released in a sea lamprey spawning stream, both the behavioral preference and the sinuosity analyses indicate that a range of mixtures is likely to be effective at drawing receiver females towards the source, provided that DkPES remains the more minor component in the mixture [22]. Minor components such as DkPES may allow receivers to gauge distance to the source, as DkPES is likely not detectable until the receiver is within proximity of a signaler. Further, we postulate that the mixture with peak behavioral activity (30:1, 3kPZS:DkPES) remains within a range similar to that released by mature males. Our previous study observed an average 30:1 ratio of 3kPZS:DkPES (10^−10^ M 3kPZS:3.3x10^-12^ M DkPES) in wash-water extracts collected from a group of mature males [22]. In the future, it will be useful to test individual male variation in ratios, and examine factors that may influence these ratios.

The increasing behavioral preference for a mixture of pheromone components that approaches that of the natural ratio released from a signaler sea lamprey is consistent with research on insects such as moths to mixtures of pheromone components [2, 6, 29]. While several components and variations of mixtures can yield attractive behaviors in moths, individual moth species often show a peak preference to the ratio that best reconstructs that of the natural ratio emitted by conspecific senders [4–6]. Behavioral data here suggests a similar relationship in sea lamprey. Unique ratios that vary between members of similar Lepidopteran species have been theorized to act as reproductive isolating mechanisms [4]. While there appears to be some overlap in sex pheromone components used among phylogenetically similar lamprey species [30], specific ratios of sex pheromones have not yet been identified in other lampreys.

The detection thresholds for the two pheromone components varied between our previous and current studies. The threshold of detection of isolated DkPES in our previous study was within a range of 10^−7^–10^−8^ M in our subjects [22], while the current study showed a EOG detection threshold of 10^−10^ M. Similarly, compound 3kPZS showed an EOG limit of detection of 10^−10^ M in the previous study [22], and 10^−13^ M in the current study. In a separate study [21], the detection threshold for 3kPZS was determined as 10^−12^ M. This variation may be attributable to sea lamprey conditions during the time of research. In our experience, longevity and sensitivity can vary based upon stream temperatures, date of capture, time held in traps by government agencies, holding conditions before transport to researchers, and length of transport. Additionally, physiological processes degrade as the spawning season progresses and sea lamprey approach natural senescence [18], which likely impacts their detection threshold of compounds and ability to respond. Finally, variability in detection thresholds may be due to an updated and more sensitive electro-olfactogram (EOG) recording system in our laboratory since our previous study and that of Siefkes et al. [21].

In summary, our data suggest that mature females detect DkPES and 3kPZS with independent receptors while making orientation decisions based on a mixture of the compounds within the odorant plume. The sinuous pattern of movement and casting into and out of the odorant plume seen here are consistent with behaviors observed in both birds and fishes when tracking odors [31]. Our data suggest that female sea lamprey encounter a plume structure, discriminate between 3kPZS and minor components such as DkPES at the olfactory level, and utilize stream flow using odor-conditioned rheotaxis [32], to gauge location of a conspecific signaler. We hypothesize that additional components or their mixtures with 3kPZS and DkPES function to arrest females inside the nest boundaries for courtship with signaler males. Taken together, examination of chemical communication in sea lamprey may provide insights into the independent detection mechanisms of pheromone mixtures at various ratios, the underlying role of these ratios as reproductive isolating mechanisms in vertebrates, and provide a useful tool for the integrated pest management of invasive sea lamprey.

## Supporting Information

S1 Table High resolution mass spectrum report for synthesized DkPES ammonium salt (HR-ESI-MS). (DOCX)

S2 Table Percentage of sexually mature female sea lamprey that moved upstream 45 m (Up) to side-by-side nest antennas activated with pheromone treatments. (DOCX)

S1 Fig DSC scan and purity analysis for the synthetic DkPES (heating rate = 1.0°C/min, sample weight = 3.20 mg). (DOCX)

S2 Fig All tracks and plumes for all treatments during field trials. Treatments and ratios are described in Fig 3.
(DOCX)

S1 Data All relevant data are within the paper and its Supporting Information files. (XLSX)
