# Supplementary material for: Mixtures of Two Bile Alcohol Sulfates Function as a Proximity Pheromone in Sea Lamprey
Source: PLoS One. 2016 Feb 17;11(2):e0149508. doi: 10.1371/journal.pone.0149508 (PMC4757539; doi:10.1371/journal.pone.0149508)
Supplement: S1 Table — (DOCX) [file pone.0149508.s001.docx]

**S1 Table.** High resolution mass spectrum report for synthesized DkPES ammonium salt (HR-ESI-MS)

Elemental Composition Report

Single Mass Analysis

Tolerance = 50.0 PPM / DBE: min = -1.5, max = 100.0

Isotope cluster parameters: Separation = 1.0 Abundance = 1.0%

Monoisotopic Mass, Odd and Even Electron Ions

13 formula(e) evaluated with 1 results within limits (all results (up to 1000) for each mass)

Minimum: -1.5

Maximum: 200.0 50.0 100.0

Mass Calc. Mass mDa PPM DBE Score Formula

449.2012 449.1998 1.4 3.1 8.5 1 C24 H33 O6 S
