# Supplementary material for: Mixtures of Two Bile Alcohol Sulfates Function as a Proximity Pheromone in Sea Lamprey
Source: PLoS One. 2016 Feb 17;11(2):e0149508. doi: 10.1371/journal.pone.0149508 (PMC4757539; doi:10.1371/journal.pone.0149508)
Supplement: S2 Table — (DOCX) [file pone.0149508.s002.docx]

**S2 Table.** Percentage of sexually mature female sea lamprey that moved upstream 45 m (Up) to side-by-side nest antennas activated with pheromone treatments.

| Treatment nest | Adjacent nest | Trials | Released | Up |
| --- | --- | --- | --- | --- |
| *3kPZS (5E-13M)* | *3kPZS (5E-13M)* | 12 | 112 | *29%* (33) A |
| *SMW* | *River water* | 8 | 81 | *33%* (27) A |
| *1:1* | *3kPZS (5E-13M)* | 9 | 90 | *28%* (25) A |
| *10:1* | *3kPZS (5E-13M)* | 7 | 69 | *29%* (20) A |
| *20:1* | *3kPZS (5E-13M)* | 6 | 66 | *23%* (15) A |
| *30:1* | *3kPZS (5E-13M)* | 7 | 81 | *19%* (15) A |
|  |  |  | X^2^ | 5.85 |
|  |  |  | df | 5 |
|  |  |  | *P*-value | 0.322 |

This table shows the percentage of subjects that moved upstream and approached the nests at the field site described in Fig 3A. The percentage that entered each treatment is shown in Fig 4. Treatments are described in Fig 3.
