# Supplementary figures and images for: Mixtures of Two Bile Alcohol Sulfates Function as a Proximity Pheromone in Sea Lamprey
Source: PLoS One. 2016 Feb 17;11(2):e0149508. doi: 10.1371/journal.pone.0149508 (PMC4757539; doi:10.1371/journal.pone.0149508)

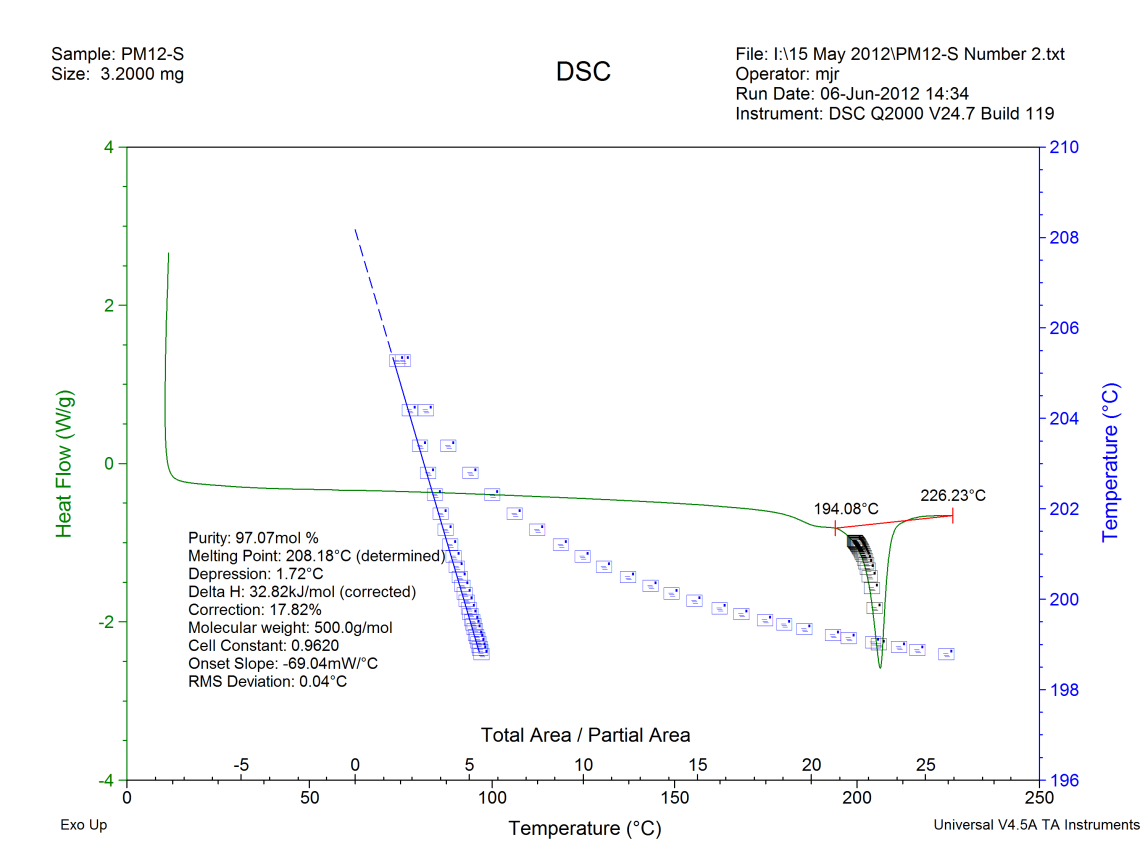


**S1 Fig.** DSC scan and purity analysis for the synthetic DkPES (heating rate = 1.0 oC/min, sample weight = 3.20 mg).

Supplement: S1 Fig — (DOCX) [file pone.0149508.s003.docx]
