# Supplementary material for: Mixtures of Two Bile Alcohol Sulfates Function as a Proximity Pheromone in Sea Lamprey
Source: PLoS One. 2016 Feb 17;11(2):e0149508. doi: 10.1371/journal.pone.0149508 (PMC4757539; doi:10.1371/journal.pone.0149508)
Supplement: S2 Fig — Treatments and ratios are described in Fig 3. (DOCX) [file pone.0149508.s004.docx]

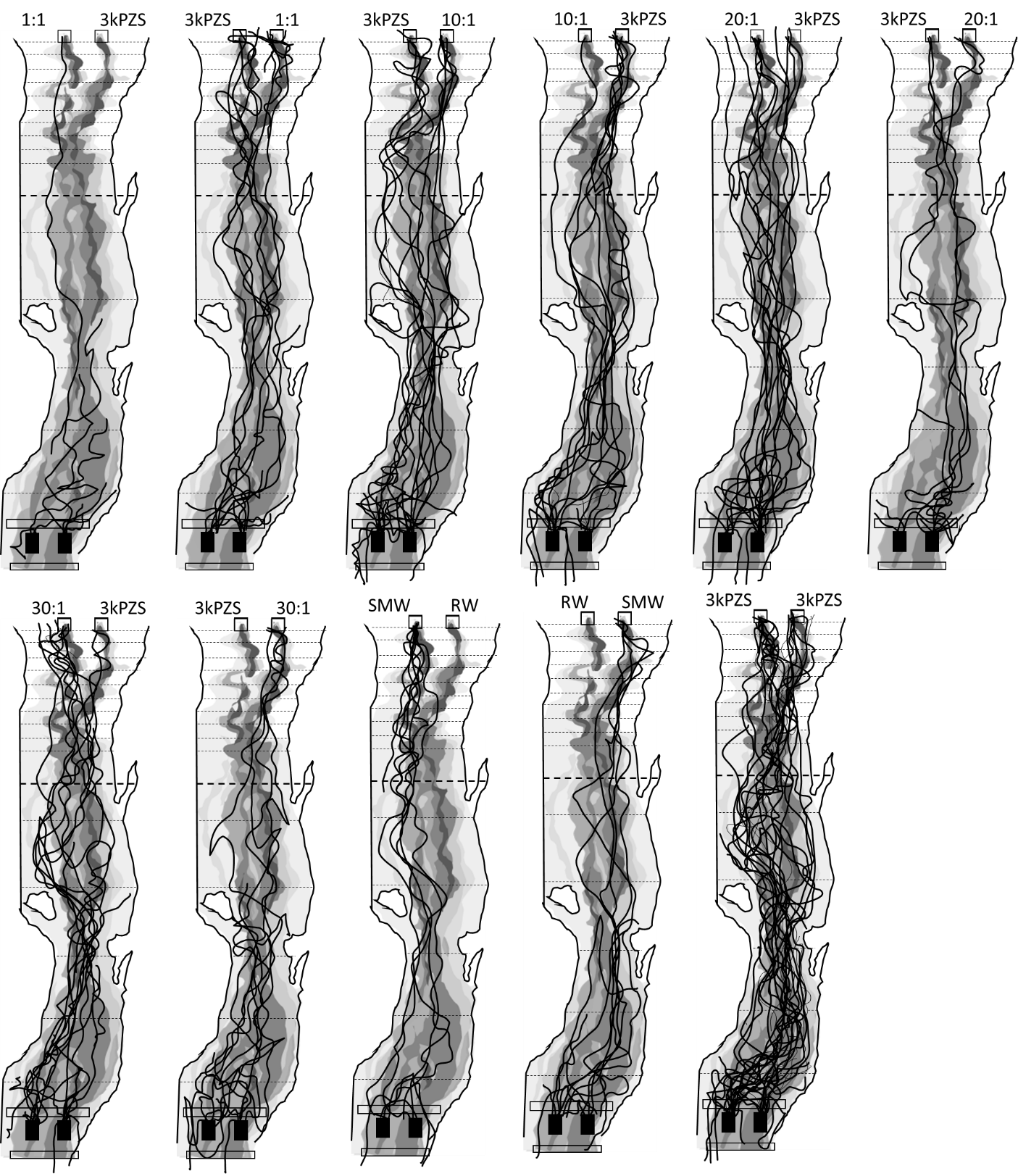


**S2 Fig.** All tracks and plumes for all treatments during field trials. Treatments and ratios are described in Fig 3.
